# Hydrophobins are required for conidial hydrophobicity and plant root colonization in the fungal biocontrol agent *Clonostachys rosea*

**DOI:** 10.1186/1471-2180-14-18

**Published:** 2014-01-31

**Authors:** Mukesh K Dubey, Dan Funck Jensen, Magnus Karlsson

**Affiliations:** 1 Uppsala BioCenter, Department of Forest Mycology and Plant Pathology, Swedish University of Agricultural Sciences, Box 7026, 75007 Uppsala, Sweden

**Keywords:** Antagonism, Biocontrol, *Clonostachys rosea*, Filamentous fungi, Gene knockout, Hydrophobins

## Abstract

**Background:**

Filamentous fungi produce small cysteine rich surface active amphiphilic hydrophobins on the outer surface of cell walls that mediate interactions between the fungus and the environment. The role of hydrophobins in surface hydrophobicity, sporulation, fruit body formation, recognition and adhesion to host surface and virulence have been reported. The aim of the present study was to characterize the biological function of hydrophobins in the fungal biocontrol agent *Clonostachys rosea* in order to understand their potential roles in biocontrol mechanisms.

**Results:**

Based on the presence of hydrophobin domains, cysteine spacing patterns and hydropathy plots, we identified three class II hydrophobin genes in *C. rosea*. Gene expression analysis showed basal expression of *Hyd1*, *Hyd2* and *Hyd3* in all conditions tested with the exception of induced *Hyd1* expression in conidiating mycelium. Interestingly, up-regulation of *Hyd1*, *Hyd2* and *Hyd3* was found during *C. rosea* self interaction compared to interactions with the fungal plant pathogens *Botrytis cinerea* or *Fusarium graminearum* in dual culture assays. Phenotypic analysis of *C. rosea* deletion and complementation strains showed that Hyd1 and Hyd3 are jointly required for conidial hydrophobicity, although no difference in mycelia hydrophobicity was found between wild type (WT) and mutant strains. Interestingly, mutant strains showed increased growth rates, conidiation and enhanced tolerances of conidia to abiotic stresses. Antagonism tests using *in vitro* dual culture and detached leaf assays showed that the mutant strains were more aggressive towards *B. cinerea*, *F. graminearum* or *Rhizoctonia solani*, and that aggression was partly related to earlier conidial germination and enhanced tolerance of mutant strains to secreted fungal metabolites. Furthermore, *in vitro Arabidopsis thaliana* root colonization assays revealed reduced root colonization ability of the Δ*Hyd3* strain, but not for the Δ*Hyd1* strain. Furthermore, enhanced root colonization ability for the Δ*Hyd1*Δ*Hyd3* strain was found in comparison to WT.

**Conclusions:**

These results show a role for hydrophobins in conidial hydrophobicity, control of conidial germination under stress conditions, and in root colonization in *C. rosea*. However, functional studies of Hyd2 remains to be performed in order to fully assess the role of hydrophobins in *C. rosea*.

## Background

Hydrophobins are small secreted proteins, produced only by filamentous fungi, which forms amphipathic layers on the outer surface of fungal cell walls [1,2]. The hydrophobic side of the amphipathic layer is exposed to the outside environment, while the hydrophilic side is directed towards cell wall polysaccharides [1,2]. Hydrophobins are characterized by the presence of eight conserved cysteine (Cys) residues in a typical pattern [1-3]. Apart from this, they show very limited amino acid sequence similarity with each other. The Cys residues form four intra-molecular disulphide bridges suggested to prevent self-assembly of the hydrophobins in the absence of a hydrophilic-hydrophobic interface [1,2]. Based on distinct hydropathy patterns and the type of layer they form, hydrophobins are divided in to two classes [1-3]. Recent bioinformatic analyses have identified an intermediate class of hydrophobins in *Trichoderma* and *Aspergillus* species [4,5]. Class I hydrophobins form amyloid-like rodlets that are highly insoluble in water, organic solvents and detergents like SDS and require strong acids for solubilisation, while amphipathic monolayers formed by class II hydrophobins lack the fibrillar rodlets and can be dissolved in aqueous organic solvents and detergents [1,2]. Another distinguishing characteristic of hydrophobins is the specific spacing patterns of conserved Cys residues; the consensus Cys spacing pattern C-X_5-10_-CC-X_33-41_-C-X_16-25_-C-X_5_- CC-X_13-17_-C of Class I differs from the consensus Cys spacing pattern C-X_9-10_-CC-X_11_-C-X_16_-C-X_8-9_- CC-X_10_-C of Class II [3-5].

Hydrophobins act as natural surfactants and reduce the surface tension of the medium, and perform a variety of biological functions in the life cycle of filamentous fungi. These include formation of a protective layer surrounding the hyphae and sexual structures, development of aerial hyphae, sporulation and spore dispersal, and fruit body formation [1-3]. In addition, hydrophobins mediate contact and communication between the fungus and its environments; that can include recognition and adhesion to host surfaces, and development of penetration structures during pathogenic and symbiotic interactions [3,6,7]. Hydrophobin MPG1 of the rice blast fungus *Magnaporthe oryzae* is necessary for leaf surface attachment and appresorium formation [8], while another hydrophobin MHP1, of the same fungus is involved in the late stage of pathogenesis [9]. In the entomopathogenic fungus *Beauveria bassiana*, deletion of hydrophobin genes results in decreased spore hydrophobicity and adhesion, loss of water-mediated dispersal, and lowered virulence to insects [10]. Similarly, in another entomopathogenic fungus, *Metarhizium brunneum*, characterization of hydrophobins showed their role in conidiation, hydrophobicity, pigmentation and virulence [11]. Contrary to these reports, functional characterization of hydrophobins in *Fusarium verticilloides* does not indicate a role of these proteins in growth, infection or mycelium hydrophobicity [12]. Similar results are reported for *Botrytis cinerea* where deletion mutants of hydrophobin genes does not display any phenotypic differences compared to the wild type (WT) strain [13].

The fungus *Clonostachys rosea* is a ubiquitous soil borne ascomycete known for its antagonistic abilities against a wide range of plant pathogens [14-18], and for its entomopathogenic and nematophagous behaviour [19-21]. The modes of action of *C. rosea* as a biological control agent (BCA) are not fully known, although mycoparasitism, competition for nutrients, and secondary metabolite production are suggested to play significant roles [14,18,22]. Furthermore, *C. rosea* can rapidly colonize outer and inner root surfaces (epidermal and cortical cells) of plants like carrot, barley, cucumber and wheat [23,24], which results in induced defence responses [25]. Hydrophobins in mycoparasitic *Trichoderma* spp, are suggested to be involved in hyphal development, sporulation, and plant root attachment and colonization [26-28].

The current study aims to understand the biological function of hydrophobins in *C. rosea* with emphasis on its role in fungal growth and development, antagonism, and interactions with plants. Our results showed induced expression of *C. rosea Hyd1*, *Hyd2* and *Hyd3* in dual cultures during self interaction in comparison to interaction with the phytopathogenic fungi *B. cinerea* and *F. graminearum*. In addition, *Hyd1* showed significant upregulation in conidiating mycelium, although a basal expression of *C. rosea Hyd1*, *Hyd2* and *Hyd3* was observed in all conditions tested. By generating individual Hyd1 and Hyd3 deletion (Δ*Hyd1*; Δ*Hyd3*), complementation (Δ*Hyd1*+; Δ*Hyd3*+) and Hyd1, Hyd3 double deletion (Δ*Hyd1*Δ*Hyd3*) strains, we probed the roles of two *C. rosea* hydrophobins in conidial hydrophobicity and plant root colonization.

## Results

### Identification and phylogenetic analysis of *C. rosea* hydrophobins

Blast searches against a *C. rosea* strain IK726 draft genome database using a total of 35 class I, class Ia (the intermediate class) and class II hydrophobin amino acid sequences from *Trichoderma* spp. [29], identified three genes with an E-value ≤ 1 × 10^-5^. The presence of additional hydrophobin gene/s in *C. rosea* genome cannot be excluded, as the short hydrophobin genes may be problematic to predict. Identification of start and stop codons, determination of exon-intron boundaries and open reading frames (ORFs) were done manually, and were further validated by cDNA sequencing. The resulting genes were named *Hyd1*, *Hyd2* and *Hyd3*. The *Hyd1*, *Hyd2* and *Hyd3* sequences are submitted to GenBank with accession numbers KF834267, KF834268, KF834269, respectively. The 267 bp *Hyd1* ORF was interrupted by a predicted 48 bp intron, the 372 bp *Hyd2* ORF was interrupted by a predicted 86 bp intron, while the 300 bp *Hyd3* ORF was interrupted by a predicted 62 bp intron. The predicted 88, 123 and 99 amino acid (aa) sequences of Hyd1, Hyd2 and Hyd3, respectively, all contained a 60-65 aa core structure that contained the Cys residues. The conserved domain analysis of translated aa sequences using Simple Modular Architecture Research Tool (SMART) identified a single hydrophobin_2 domain (Pfam 06766) between aa positions 21-86, 21-85 and 30-91for Hyd1, Hyd2 and Hyd3, respectively. This structure was further confirmed by InterproScan and Conserved Domain Search (CDS) analyses. Signal P predicted 16-18 aa long secretion signal peptides in the N-termini of each *C. rosea* hydrophobin. The highest similarity of Hyd1 was with cerato-ulmin of *Geosmithia* spp. and *Ophistoma nova-ulmi* (e-value 3e-07; identity 33%), of Hyd2 with *T. atroviride* hydrophobin and spore related hydrophobin of *T. viride* (e-value 3e-10; identity 41%), and of Hyd3 with hydrophobin from *Fusarium* spp. (e-value 3e-32; identity 73%). In addition, aa similarity between Hyd1, Hyd2 and Hyd3 were below 20%.

**Figure 1:**
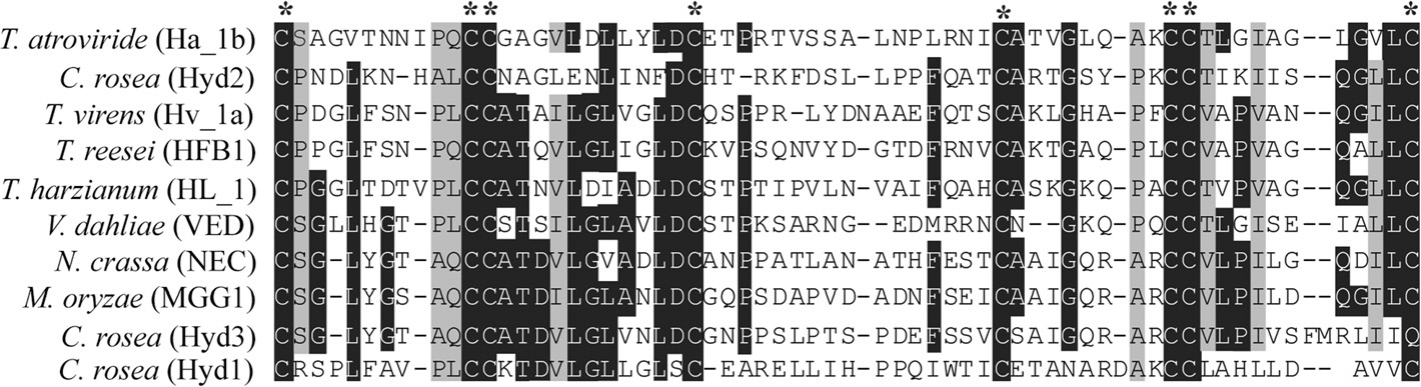
**Sequence alignment of *C*. *rosea *hydrophobins.** Amino acid sequence alignment of *C. rosea* hydrophobins with class II hydrophobins from *Trichoderma* spp. and additional representatives of known class II hydrophobins. The amino acid sequences from first Cys to eight Cys residues were used for the alignment. Conserved residues in a column are indicated in white and boxed in black; two different conserved residues in a column are highlighted by grey boxes; gaps are indicated by dashes. Conserved Cys residues are indicated by asterisks.

Hyd1 and Hyd2 contained eight Cys in their protein sequences, while Hyd3 contained only seven as the Cys residue closest to the C-terminus was replaced by a glutamine (Gln) (Figure 1). This replacement was similar to the *T. harzianum* hydrophobin QID3 that also contained seven Cys [30], although Hyd3 did not show the extended N-terminus of QID3. The Cys spacing of Hyd1, Hyd2 and Hyd3 conformed to the pattern of Class II (Figure 1). Furthermore, the hydropathy patterns of Hyd1, Hyd2 and Hyd3 were all indicative of class II hydrophobins (data not shown). Taken together, these analyses suggest that *C. rosea* Hyd1, Hyd2 and Hyd3 encode putative class II hydrophobins.

**Figure 2:**
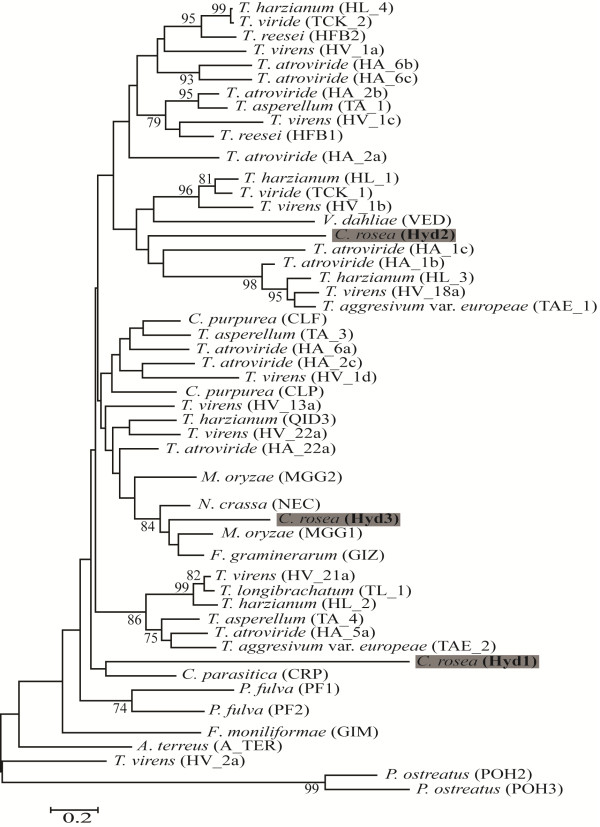
**Phylogenetic analysis of *C*. *rosea *hydrophobins.** Phylogenetic analysis of class II hydrophobins using maximum likelihood methods implemented in PhyML-aBayes. *Pleurotus ostreatus* hydrophobins are used as out group. Branch support values (bootstrap proportions ≥ 70%) are associated with nodes. The bar marker indicate the number of amino-acid substitutions.

A phylogenetic tree was constructed with Hyd1, Hyd2 and Hyd3 together with class II hydrophobins from *Trichoderma* spp. and additional representatives of known class II hydrophobins (Additional file 1: Table S1). The result from the phylogenetic analysis showed that Hyd1, Hyd2 and Hyd3 do not represent recent gene duplicates as they clustered in different parts of the tree (Figure 2).

### Expression analysis of *Hyd1*, *Hyd2* and *Hyd3*

**Figure 3:**
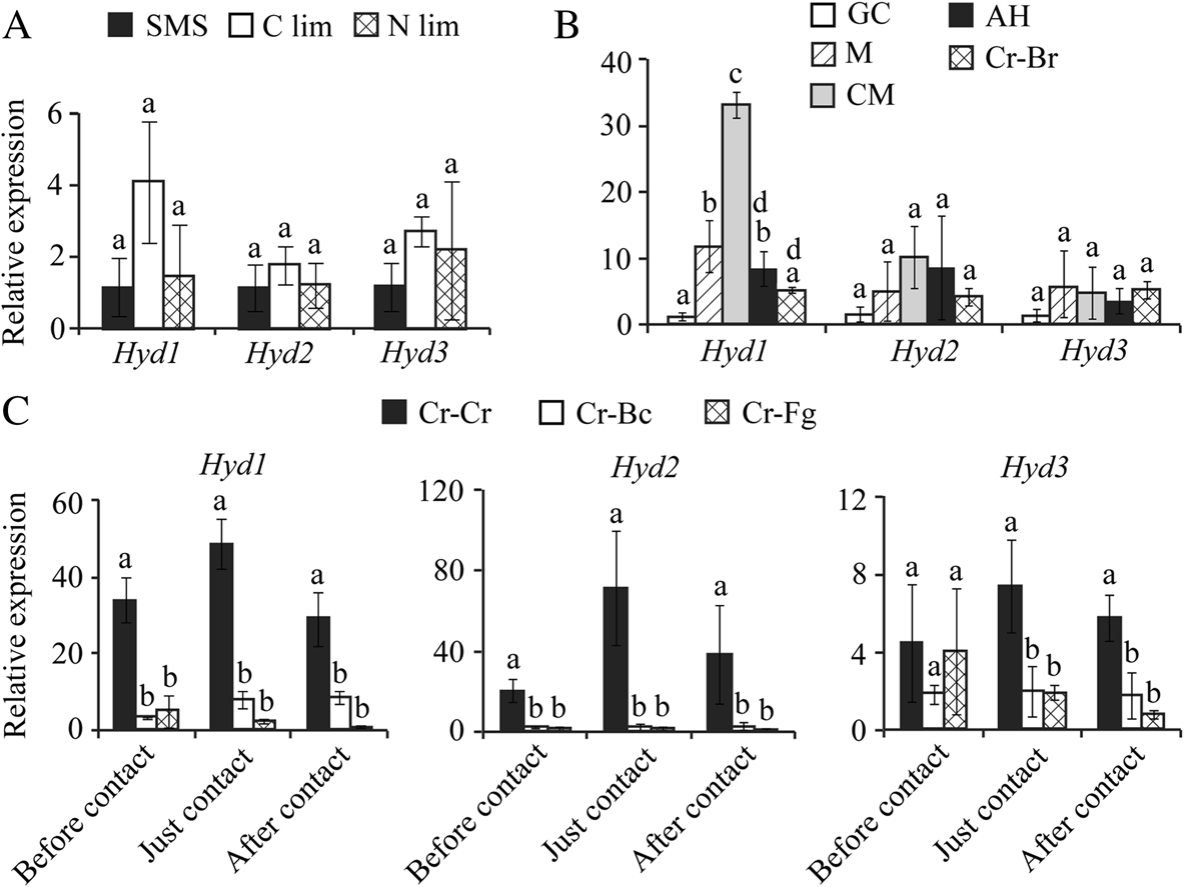
**Expression analyses of hydrophobin genes in *C*. *rosea*. A:** Total RNA was extracted from mycelia 24 h post incubation in submerged shake flask culture in glucose, C lim and N lim medium. **B:** Total RNA was extracted from mycelia of different developmental stages like germinating conidia (GC), vegetative mycelium (M), Conidiated mycelim (CM), aerial hyphae (AH) and post five days interaction with barley roots (Cr-Br). **C:** gene expression analysis during different stages of interaction with *B. cinerea* (Cr-Bc) or *F. graminearum* (Cr-Fg). *C. rosea* confronted with itself was used as control (Cr-Cr). Expression levels for *Hyd1*, *Hyd2* and *Hyd3* was normalized by tubulin expression, using the formula described by Pfaffl [52]. Error bars represent standard deviation based on 3 biological replicates. Different letters indicate statistically significant differences (*P* ≤ 0.05) within experiments based on the Tukey-Kramer test.

Quantitative PCR (qPCR) was used to analyse the expression pattern of *C. rosea* hydrophobins. In relation to glucose, no significant expression changes in *Hyd1*, *Hyd2* or *Hyd3* expression were found in SMS culture representing carbon limitation (C lim) or nitrogen limitation (N lim) (Figure 3A). Gene expression analysis was performed on RNA extracted from germinated conidia (GC), mycelium (M), conidiating mycelium (CM), aerial hyphae (AH), and during interaction with barley roots (Cr-Br). In relation to GC, a significant (*P* ≤ 0.03) induction in *Hyd1* expression was found in M, CM and AH (Figure 3B). In addition, CM showed significant (*P* = 0.03) induced expression of *Hyd1* in comparison with M, AH and Cr-Br (Figure 3B). No significant changes in expression of *Hyd2* or *Hyd3* were found in any of the developmental conditions tested or during root interaction (Figure 3B). For hydrophobin gene expression during interactions between *C. rosea* and *B. cinerea* or *F. graminearum*, RNA was extracted from the mycelium harvested at different stages of interaction as described in methods section. Transcript levels of *C. rosea* hydrophobins were found to be significantly induced (*P* ≤ 0.013) at all stages of self interaction in comparison with interspecific interactions (Figure 3C). No significant difference in expression of *C. rosea* hydrophobin genes were found between different stages of interaction with either of prey fungus except the significant (*P* ≤ 0.02) induced expression of *Hyd1* at contact and after contact stage in comparison to before contact stage during the interaction with *B. cinerea*, but not with the *F. graminearum* (Additional file 1: Figure S1). An additional observation was that a basal expression of all *C. rosea* hydrophobin genes was observed in all tested conditions.

### Generation of *Hyd1*, *Hyd3* and *Hyd1Hyd3* deletion and complementation strains

Single *Hyd1* and *Hyd3* deletion mutants were generated by replacing *Hyd1*and *Hyd3* with the hygromycin resistance gene selection cassette (*hygB*) by *Agrobacterium tumefaciens* mediated transformation (ATMT). A double Δ*Hyd1*Δ*Hyd3* deletion strain was constructed by replacing *Hyd3* with the nourseothricin resistance gene selection cassette (*nat1*) in a Δ*Hyd1* strain. Despite several attempts of transformation and screening of more than 200 hygromycin resistance colonies, we failed to generate a *Hyd2* deletion mutant. Successful gene replacement in mitotically stable putative mutants was confirmed by PCR as described previously [31-33] using primers located within the *hygB/nat1* cassettes together with primers located upstream and downstream of the construct (Additional file 1: Figure S2A, E, I). The expected size of PCR fragments were amplified in Δ*Hyd1*, Δ*Hyd3* and Δ*Hyd1*Δ*Hyd3* strains, while no amplification was observed in wild type (WT) (Additional file 1: Figure S2B, F, J). The complete deletion of *Hyd1*and *Hyd3* was further confirmed by PCR amplification of fragments of expected size using primer pairs located outside the construct borders, from mutant and WT strains (Additional file 1: Figure S2B, F, J). Furthermore, reverse transcriptase PCR (RT-PCR) experiments using primers specific to *Hyd1*and *Hyd3* sequences demonstrated the complete loss of *Hyd1* and *Hyd3* transcript in each individual and double deletion mutants (Additional file 1: Figure S2C, G, K).

Single *Hyd1* and *Hyd3* deletion mutants were complemented with WT *Hyd1* and *Hyd3* genes respectively, through ATMT. Successful integration of the Hyd1-comp and Hyd3-comp vectors (including the *nat1* selection cassette) in mitotically stable mutant was confirmed by PCR amplification of *nat1* (data not shown). RT-PCR from randomly selected *nat1* positive *Hyd1* and *Hyd3* complemented (Δ*Hyd1*+; Δ*Hyd3+*) strains using *Hyd1*- and *Hyd3-*specific primer pairs demonstrated restored *Hyd1* and *Hyd3* transcription while no transcripts were detected in the parental deletion strains (Additional file 1: Figure S2D, H).

### Effects of *Hyd1* and *Hyd3* deletion on colony morphology, growth rate, conidiation, hydrophobicity, and secreted protein concentration

**Figure 4:**
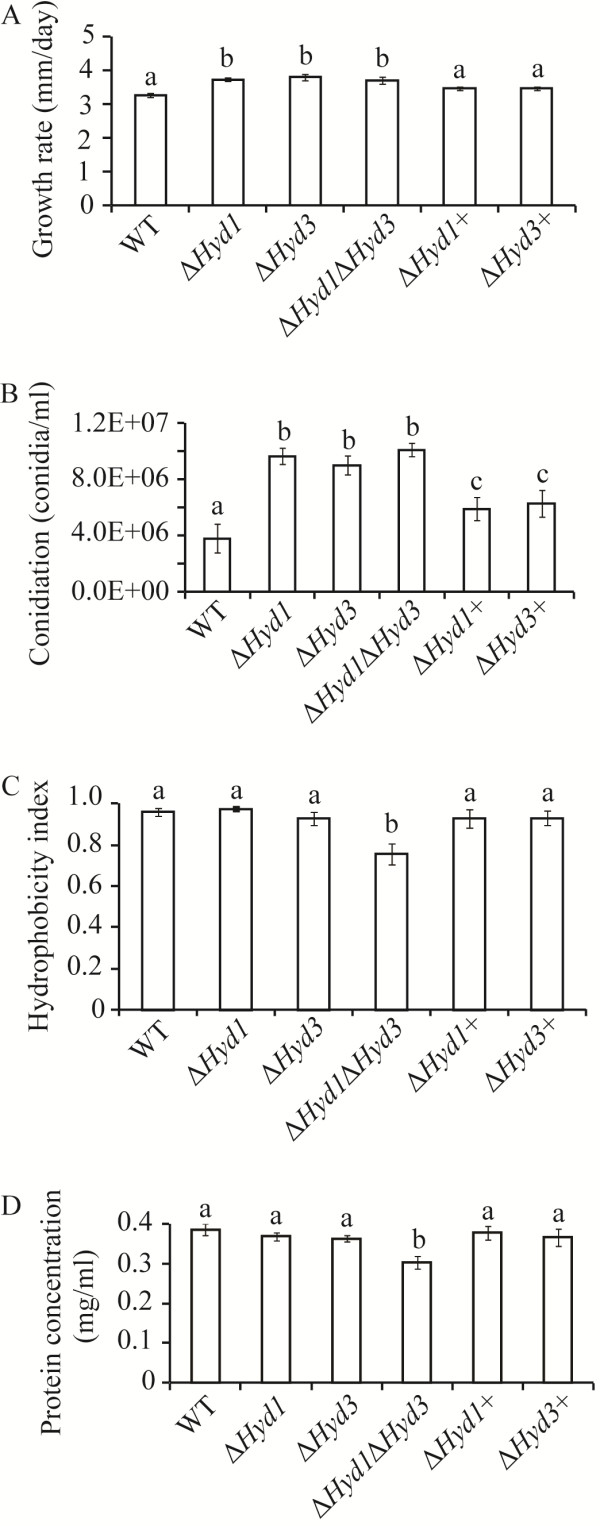
**Phenotypic characterizations of *C*. *rosea *hydrophobin mutants. A:** Growth rate of WT, mutants and complemented strain on PDA medium. Strains were inoculated on solid agar medium, incubated at 25°C and the growth diameter was recorded 5 days post inoculation. **B:** Conidiation of WT, mutants and complemented strain on PDA medium 10 days post inoculation. Conidia were harvested in equal volume of water and number was determined using a Bright-Line haemocytometer as per instruction of manufacturer. **C:** Cell surface hydrophobicity of WT, deletions and complemented strains conidia as determined by microbial adhesion to hydrocarbon (MATH) assay. **D:** Total extracellular protein concentration of WT deletions and complemented strains. Culture filtrates of 10 days grown fungal strains were used for protein precipitation. Error bars represent standard deviation based on 3 biological replicates. Different letters indicate statistically significant differences (*P* ≤ 0.05) based on the Tukey-Kramer test. Experiments were repeated two times with same results.

No difference in colony morphology was found between WT and deletion mutants (data not shown). All deletion strains showed significantly (*P* < 0.001) increased growth rate and conidiation on potato dextrose agar (PDA) medium in comparison to WT, although no differences were detected between single deletion strains or between single and double deletion strains (Figure 4A, B). Complementation strains Δ*Hyd1*+ Δ*Hyd3+* showed partial restoration of normal conidiation levels (Figure 4B).

Hydrophobicity of WT and mutant strains were tested by carefully placing 10 μl water or SDS (0.2% or 0.5%) droplets onto the surface of non-conidiating mycelia (3 days post inoculation on PDA). All droplets remained on the surface of mycelium and no visible difference in shape or contact angle of droplets was found in between WT and mutant strains even up to overnight incubation in closed Petri-dishes at room temperature. Similar results were obtained when conidiated mycelia (10 days post inoculation) were used. Conidial surface hydrophobicity was further analysed by using an assay for microbial adhesion to hydrocarbons (MATH) [34]. The MATH assay showed no difference in hydrophobicity index between WT and single deletion mutants; however conidia of the double deletion mutant showed significant (*P* < 0.001) reduction in hydrophobicity index (Figure 4C). In addition, unlike the WT, Δ*Hyd1* and Δ*Hyd3*, conidia from the Δ*Hyd1*Δ*Hyd3* strain formed cell aggregates when harvested in water (Additional file 1: Figure S3).

To analyse total protein secretion, protein concentrations were determined in culture filtrates of WT and mutant strains grown in liquid potato dextrose broth (PDB) medium. Results showed a significant (*P* ≤ 0.004) 9% reduction in protein concentration in Δ*Hyd1*Δ*Hyd3* culture filtrates compared to WT or single deletion strains, while no differences were observed in between WT and Δ*Hyd1* or Δ*Hyd3* strains (Figure 4D).

### Effect of *Hyd1* and *Hyd3* deletion on abiotic stress tolerance

**Figure 5:**
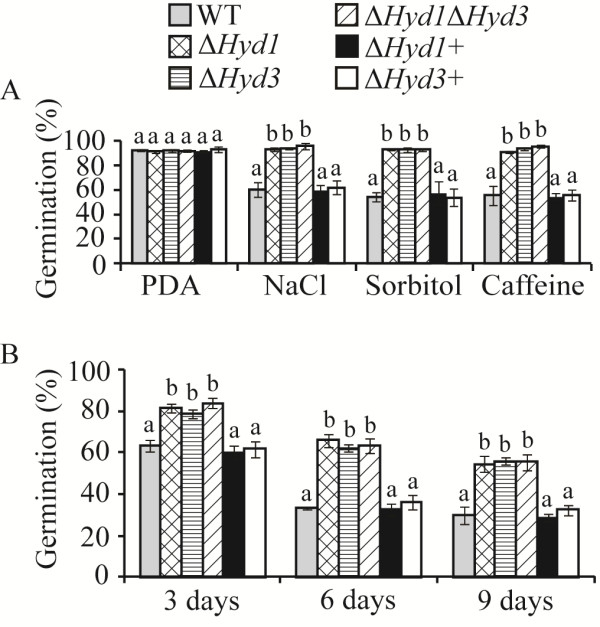
**Abiotic stress tolerances of *C*. *rosea *WT and mutant strains. A:** Frequency of conidia germination on medium containing NaCl, sorbitol, SDS, or caffeine as abiotic stress agents. Conidia spread on PDA plate were served as control. **B:** Frequency of conidia germination after cold shock at 4°C for 3 days, 6 days or 9 days. *C. rosea* WT, mutants and complementation strains conidia were spread on agar plates and frequency of conidial germination was determined by counting two hundred to three hundred conidial germ-tubes or conidia under microscope for each treatment. Each experiment was repeated two times. Error bars represent standard deviation based on 3 biological replicates. Different letters indicate statistically significant differences (*P* ≤ 0.05) based on the Tukey-Kramer test.

Susceptibility of WT and mutant strains to various abiotic stress conditions were tested on PDA plates containing NaCl, sorbitol, SDS, or caffeine. No significant differences in growth rate were recorded between mutant and WT strains on any of the tested stress media, except for significantly (*P* = 0.028) increased growth rate of the double deletion mutant Δ*Hyd1*Δ*Hyd3* on PDA containing NaCl (Additional file 1: Figure S4). Significant (*P* < 0.001) increases in conidial germination rates (> 90%) were recorded in mutant strains in comparison with WT (55% to 60%) on all tested abiotic stress media, although no differences were found between WT and mutant strains on control PDA medium (Figure 5A). In another set of experiments we assayed the conidial susceptibility to cold. After 3, 6 or 9 days of incubation at 4°C, only 63%, 33% or 30% respectively, of WT conidia germinated after placing them on PDA medium for16 h at 25°C. In contrast, similarly treated conidia of mutants strain showed significantly (*P* < 0.001) higher germination rates (82%, 64% and 56%) (Figure 5B). However, no differences in conidial germination between either of single or double deletion mutants were found in any of the stress condition tested (Figure 5).

### Deletion of *Hyd1* and *Hyd3* did not affect *Hyd2* expression

In order to examine whether or not deletion of *Hyd1* and *Hyd3*, individually or simultaneously, affects the expression pattern of *Hyd2*, RNA was extracted from conidiating mycelium of WT and mutant strains grown on PDA plates. Gene expression analysis revealed no significant difference in *Hyd2* expression between WT and either single or double deletion strains (Additional file 1: Figure S5).

### *In vitro* assay to test the antagonistic ability of *C. rosea* strains

**Figure 6:**
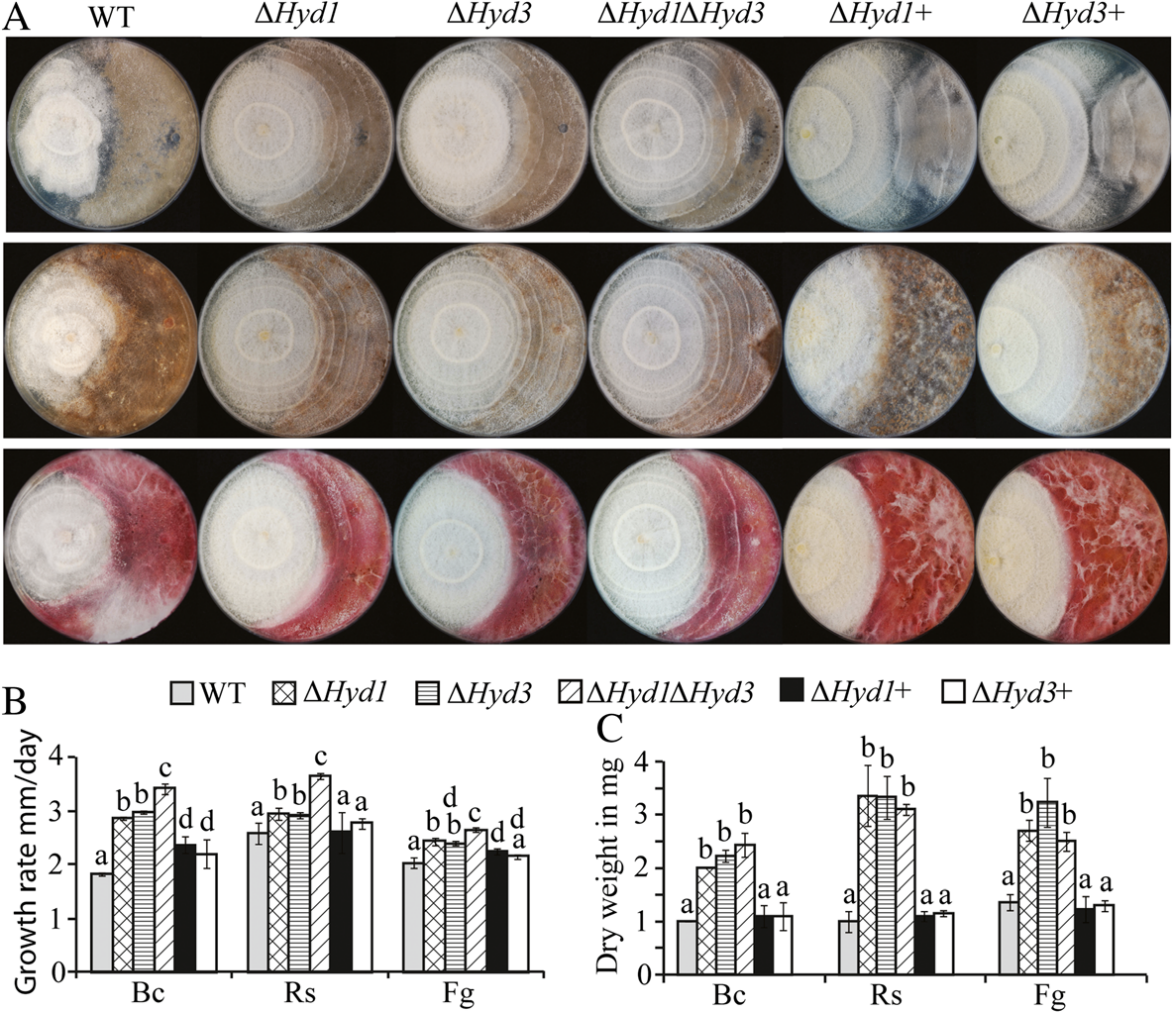
**Antagonism analyses of *C*. *rosea *strains. A:** Plate confrontation assay against *B. cinerea* (Uppar lane), *R. solani* (middle lane) and *F. graminearum* (lower lane). Agar plugs of *C. rosea* (left side in the plate) strains and B*. cinerea*, *R. solani* or *F. graminearum* (right side in the plate) were inoculated on opposite sides in 9 cm diameter agar plates and incubated at 25°C. The experiment was performed in 3 replicates and photographs of representative plates were taken 20 days post inoculation. **B:** Tolerance of *C. rosea* strains to the secreted metabolites of *B. cinerea* (Bc), *R. solani* (Rs) and *F. graminearum* (Fg). Agar plugs were inoculated on PDA plates covered with cellophane and incubated at 25°C in darkness. After reaching to the end of plate the colony was removed together with the cellophane disc. Plates were re-inoculated with a *C. rosea* WT, Δ*Hyd1*, Δ*Hyd3*, Δ*Hyd1*Δ*Hyd3*, and Δ*Hyd1*+, Δ*Hyd3*+ agar plug, incubated at 25°C and linear growth was recorded daily. **C:** Secretion assay of *C. rosea* strain. Fungal strains were grown in potato dextrose broth for 10 days at 25°C. Culture filtrates was collected after removing mycelia mass and were inoculated with *B. cinerea* (Bc), *R. solani* (Rs) or *F. graminearum* (Fg) agar plug. Biomass production in culture filtrates was analysed by determining mycelial dry weight post 3 days of inoculation. Error bars represent standard deviation based on 3 biological replicates. Different letters indicate statistically significant differences (*P* ≤ 0.05) within experiments based on the Tukey-Kramer test.

The Δ*Hyd1*, Δ*Hyd3*, and Δ*Hyd1*Δ*Hyd3* strains overgrew *B. cinerea*, *F. graminearum* and *Rhizoctonia solani* faster than the WT in plate confrontation assays (Figure 6A). The complemented strains Δ*Hyd1*+ Δ*Hyd3*+ showed partial restoration of WT behaviour. Furthermore, in order to understand the tolerance of *C. rosea* strains to the secreted metabolites from the fungal prey, a secretion assay was performed. Growth rates of deletion strains were significantly (*P* < 0.001) higher than the WT when grown on agar plates where *B. cinerea*, *F. graminearum* or *R. solani* were pregrown (Figure 6B). In addition, the double deletion strain Δ*Hyd1*Δ*Hyd3* showed significantly (*P ≤* 0.05) higher growth rate compared to the either single deletion mutant (Figure 6B). Similarly to the plate confrontation assay, Δ*Hyd1*+ and Δ*Hyd3*+ strains showed partial restoration of WT growth rates.

In another set of experiments, mycelial biomass of *B. cinerea*, *F. graminearum* and *R. solani* was measured in sterile-filtered culture filtrates of *C. rosea* WT and deletion strains. A significantly (*P* < 0.001) higher biomass production of *B. cinerea*, *F. graminearum* and *R. solani* was recorded when grown in culture filtrates of hydrophobin deletion strains compared with WT culture filtrate (Figure 6C). No differences in fungal biomass production were found between culture filtrates of either single or double mutant strains (Figure 6C).

### Assessment of antagonistic activity of *C. rosea* strains using a detached leaves assay

**Figure 7:**
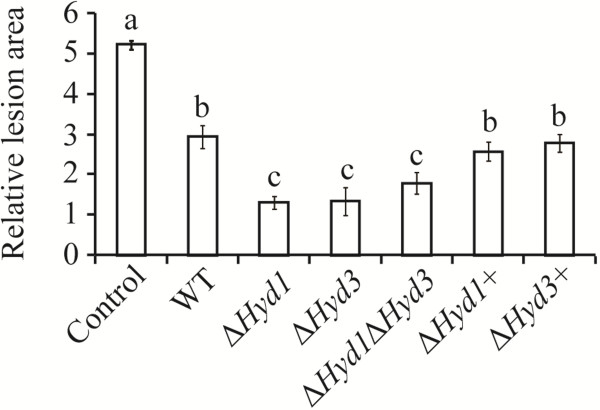
**Measurement of *B*. *cinerea *necrotic lesions on detached leaves of *A. thaliana *plants.** The leaves were inoculated with *C. rosea* strains 30 minute before application of *B. cinerea* and allowed to interact for 56 h. Only pathogen inoculated leaves were used as control. Necrotic lesion area was measured under the microscope using DeltaPix camera and software. Error bars represent standard deviation based on 3 biological replicates. Different letters indicate statistically significant differences (*P* ≤ 0.05) within experiments based on the Tukey-Kramer test.

A significant (*P* < 0.001) reduction in necrotic lesion area was measured on leaves preinoculated with *C. rosea* WT compared to control leaves where only *B. cinerea* was inoculated (Figure 7). In addition, in leaves preinoculated with Δ*Hyd1*, Δ*Hyd3*, or Δ*Hyd1*Δ*Hyd3* strains, necrotic lesion areas were significantly (*P* < 0.001) less severe than those observed in WT preinoculated leaves. No difference in necrotic lesion areas were found between leaves preinoculated with either single or double deletion strains (Figure 7).

### Assessment of *C. rosea* strains for root colonization ability

**Figure 8:**
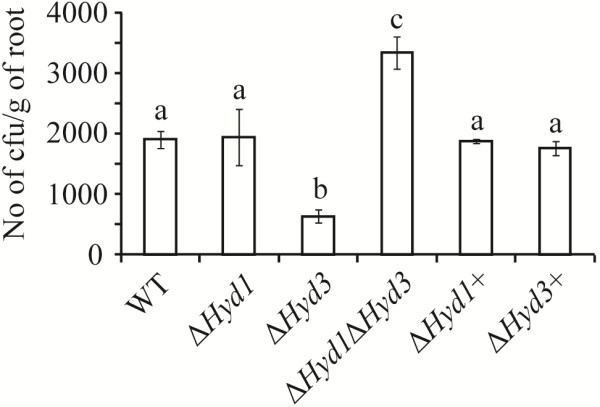
***A. thaliana *root colonization by *C. rosea *strains.***A. thaliana* roots were detached 5 days post inoculation and washed. After sterilization in 2% NaOCl for 1 min, the roots were homogenized in water and serial dilutions were plated on PDA plates under sterile condition at 25°C. Different letters indicate statistically significant differences (*P* ≤ 0.05) based on the Tukey-Kramer test.

*Arabidopsis thaliana* roots, grown on MS plates, were inoculated with *C. rosea* conidia and allowed to interact for 5 days. Water inoculated roots were used as control. After surface sterilization, colonization levels were determined by counting colony forming units (cfus). No significant differences in root colonization ability were recorded between WT and the Δ*Hyd1* strain. In contrast, root colonization by the Δ*Hyd3* strain was significantly (*P* < 0.001) reduced (Figure 8). Interestingly, the double deletion Δ*Hyd1*Δ*Hyd3* strain showed increased (*P* < 0.001) colonization ability compared to WT or single deletion strains (Figure 8).

## Discussion

Filamentous fungi generally contain multiple hydrophobin genes, which play important roles in fungal growth, development and environmental communication [1,2,6,7]. We identified only 3 class II hydrophobin genes in the genome of the mycoparasite *C. rosea*. This is in strong contrast with the closely related mycoparasites *T. atroviride* and *T. virens* that contain high numbers (10 and 9 respectively) and diversity of class II hydrophobins [29]. This indicate important ecological differences between *C. rosea* and *Trichoderma* spp., and emphasize that different mycoparasites may rely on different mechanisms of interaction. The expansion of the hydrophobin gene family in *Trichoderma* spp. is hypothesized to help the fungus to attach to the hyphae of a broad range of asco- and basidiomycetes [29].

The high expression of *Hyd1* in conidiating mycelia in comparison with germinating conidia indicates that Hyd1 may have a role during conidiophore development. This is consistent with the expression pattern of *hyd1* in *M. anisoplia* where expression is low in germinating conidia and high in mycelium with conidiophores [35]. The expression, but lack of regulation, of *Hyd1*, *Hyd2* and *Hyd3* on different nutrient regimes, and between developmental stages of *Hyd2* and *Hyd3*, indicate a constitutive role of the corresponding proteins in *C. rosea*. Constitutive roles of hydrophobins in fungal growth and development are reported in many species [6,7,36]. However, certain hydrophobins from *Trichoderma* spp. and *M. brunneum* are regulated by nutritional conditions and between different life cycle stages [5,11,28,37].

Expression levels of *Hyd1*, *Hyd2* and *Hyd3* are repressed in *C. rosea* during interactions with *B. cinerea* and *F. graminearum*, which is consistent with the expression pattern of *T. atroviride* hydrophobin genes *hfb-1b*, *hfb-2c* and *hfb-6a*[37]. This may suggest that Hyd1, Hyd2 and Hyd3 are not involved in protecting hyphae from recognition by other organisms [6,7]. Alternatively, the data can also be interpreted as an induction during *C. rosea* self interaction that may suggest a role for Hyd1, Hyd2 and Hyd3 in intraspecific signalling or hyphal fusion. Hydrophobins that are known to be involved in interactions with plant leaves and roots are usually highly expressed during these conditions [8,9,28]. Therefore, the low expression of the 3 *C. rosea* hydrophobin genes during barley root colonization indicates that the corresponding proteins may not be necessary for root adhesion and colonization.

Deletion of hydrophobin genes from different fungal species often results in variable and sometimes contradicting phenotypes. This is a reflection of the birth-and-death type of evolution of the hydrophobin gene family [29], which results in functionally diverse proteins with many species specific members. This is evident for Hyd1 and Hyd3 in *C. rosea* as gene deletions results in increased growth rate and sporulation, which is in contrast to the reduced sporulation in *T. reesei*, *M. oryzae* and *M. brunneum* due to deletion of the hydrophobin genes *HFB2*[26], *MPG1* and *MHP1*[8,9] and *hyd1*, *hyd2* and *hyd3*[11], respectively. The situation is even more complicated as deletion of *HCf-1* and *HCf-2* in *Cladosporium fulvum*[34], *cpph1* in *Claviceps purpurea*[38] and *hfb1* in *T. reesei*[26] results in no differences in sporulation in comparison with the WT strain.

Deletion of *Hyd1* or *Hyd3* does not influence mycelial hydrophobicity in *C. rosea*, which is consistent with previous reports in *C. purpurea*, *M. brunneum, F. verticilloides* and *B. cinerea*[11-13,38]. However, it seems that Hyd1 and Hyd3 are jointly required for conidial hydrophobicity and dispersal, as the conidia from the double deletion mutant Δ*Hyd1*Δ*Hyd3* clump together in solution and have lower hydrophobicity index than the WT. Similar phenotypes are repeatedly reported from many different species [8,9,11,12,34,39]. Furthermore, deletion of *Hyd1* and *Hyd3* does not influence the expression levels of *Hyd2*, which suggests that *Hyd2* is subject to different regulatory signals than *Hyd1* and *Hyd3*. Failure to delete *Hyd2* despite several trials may suggest an essential function of the corresponding protein.

Hyd1 and Hyd3 do not appear to be involved in protection of the *C. rosea* mycelium during abiotic stress conditions. In contrast, higher conidial germination rates during abiotic stress conditions in *Hyd1* and *Hyd3* mutants suggests that these hydrophobins inhibit conidial germination in environments not suitable for mycelial growth. Similar results are shown previously in *M. oryzae* and the entomopathogenic fungus *B. bassiana* against thermal stress [9,10]. Hence, under unfavourable conditions hydrophobins may act as a sensor for the conidial germination signalling pathway and consequently protect the conidia by limiting its germination until favourable conditions are prevail [10].

The increased growth rate of *Hyd1* and *Hyd3* deletion strains under normal conditions such as PDA, may explain the faster overgrowth of the fungal prey in plate confrontations and the higher growth rate on plates previously colonized by *B. cinerea*, *F. graminearum* or *R. solani*. Similar results are reported previously in *T. asperellum*, where deletion of *TasHyd1* does not reduce *in vitro* mycoparasitic ability [28]. Hydrophobins are highly expressed proteins that may account for up to 10% of the total amount of secreted proteins [40,41]. In *C. rosea*, deletion of both *Hyd1* and *Hyd3* results in a reduction of the total amount of secreted proteins. Despite this, no differences in pathogen biomass production in sterile filtered culture filtrates from single and double deletion strains are recorded. This may suggest that Hyd1 and Hyd3 do not exert a direct toxic effect on the fungal prey.

The higher conidial germination rates (under certain conditions) and higher growth rates of *Hyd1* and *Hyd3* deletion strains may explain the reduced necrotic lesion area, caused by *B. cinerea*, on *A. thaliana* leaves preinoculated with the mutant strains in comparison with WT preinoculated leaves. As a consequence, the *C. rosea* deletion strains may parasitize *B. cinerea* to a greater extent or simply outcompete it for space or nutrients. Hydrophobins in *T. asperellum* are reported to influence root surface attachment and intercellular root colonization [28]. Similarly, our results show that Hyd3 is needed for barley root colonization. Unexpectedly, deletion of *Hyd1* in a Δ*Hyd3* background increases the root colonization ability. The exact mechanism responsible for this cannot be discerned based on the current data, but we may speculate that it can be related to the lower conidial hydrophobicity or the lower protein secretion of the double deletion strain compared with the *Hyd1* and *Hyd3* single gene deletion strains. In the entomopathogenic fungus *B. bassiana*, reduced virulence is recorded for a Δ*hyd1* strain, while no effect is observed for a Δ*hyd2* strain. However, the effect of the Δ*hyd1*Δ*hyd2* double deletion mutant on virulence is cumulative and lower than for the single Δ*hyd1* strain [10].

## Conclusions

We identified three class II hydrophobin genes and characterized their function in the fungal biocontrol agent *C. rosea*. Our results showed a basal expression of all three hydrophobin genes during growth and development and under nutritional stress conditions, although *Hyd1* was induced during conidiation. In addition, all three genes were upregulated during self-interaction compared to the interaction with fungal prey. Deletion of *C. rosea Hyd1* and *Hyd3* demonstrate the involvement of the corresponding proteins in controlling conidial germination under unfavourable conditions, and the additive contribution of Hyd1 and Hyd3 to conidial hydrophobicity. Hyd3 was further shown to influence the root colonization ability of *C. rosea*.

## Methods

### Fungal strains and culture conditions

*C. rosea* strain (WT) and mutants derived from it, *B. cinerea* strain B05.10, *R. solani* strain SA1 and *F. graminearum* strain 1104-14 were maintained on PDA (Oxoid, Cambridge, UK) medium at 25°C. SMS medium [31] supplemented with 1% glucose was used for gene expression unless otherwise specified. Starvation for carbon (C lim), nitrogen (N lim) and carbon + nitrogen (C + N lim) was induced as described before [31]. *C. rosea* mycelia for submerged liquid cultures were cultivated and harvested as described previously [31].

### Gene identification and sequence analysis

The *C. rosea* strain draft genome (Karlsson et al., unpublished) was screened for the presence of hydrophobins by BLASTP analysis using amino acid sequences of *T. aggresivum* var. *europeae*, *T. asperellum*, *T. atroviride*, *T. harzianum*, *T. longibrachatum*, *T. stromaticum*, *T. virens* and *T. viride* hydrophobins. The protein accession numbers of hydrophobins from *Trichoderma* spp. (Additional file 1: Table S1) were retrieved from Kubicek et al. [29], and their amino acid sequences were retrieved from GenBank at NCBI. Presence of conserved domains were analysed with SMART [42], InterProScan [43] and CDS [44]. Presence of Cys residues in specific spacing pattern was analysed manually. Amino acid sequence alignment was performed using clustalW2 [45] with default settings for multiple sequence alignment. Signal P 4.1 [46] was used to search for signal peptide cleavage sites. Hydropathy pattern was determined with Protscale on the ExPASy proteomics server [47], using the Kyte-Doolittle algorithms and 9 aa sliding window. We generated the hydropathy pattern of Hyd1, Hyd2 and Hyd3 and compared to the hydropathy patterns of known class I (SC3 [AAA96324] from *Schizophyllum commune*; EAS [AAB24462] from *Neurospora crassa*; RodA [AFUA_5G09580] from *Aspergillus fumigatus*) and known class II (HFB1 [CAA92208.1] and HFBII [P79073] from *T. reesei*; CRP from *Cryphonectria parasitica* [AAA19638]) hydrophobins. The presence of conserved hydrophobin domains, Cys residues in a specific pattern, presence of signal peptide, and hydropathy plot were used as criteria for identification of hydrophobins in *C. rosea*.

### Phylogenetic analysis

Phylogenetic analysis was performed using maximum likelihood methods implemented in PhyML-aBayes [48]. The LG amino-acid substitution model [49] was used, the proportion of invariable sites was set to 0, and four categories of substitution rates were used. The starting tree to be refined by the maximum likelihood algorithm was a distance-based BIONJ tree estimated by the program. Statistical support for phylogenetic grouping was assessed by bootstrap analysis using 1000 replicates. GenBank accession numbers for hydrophobin proteins used in this study for phylogenetic analysis are given in Additional file 1: Table S1.

### Gene expression analysis

For gene expression analysis in different nutritional conditions (described above), mycelia were cultivated in liquid cultures following the procedure described before [31] and harvested 48 h post inoculation. SMS agar plates were used for gene expression analysis during different developmental stages and during interaction, except for conidial germination where liquid SMS medium was used. Developmental stages included M (mycelia harvested three days post inoculation), CM (mycelia harvested 10 days post inoculation), AH, and GC (24 h post inoculation of conidia in liquid SMS). For interactions, *C. rosea* was confronted with *B. cinerea* (Cr-Bc) or *F. graminearum* (Cr-Fg) on agar plates and the growing front (7-10 mm) of *C. rosea* was harvested before contact (5-7 mm apart), at contact, and post 24 h contact. *C. rosea* confronted with itself (Cr-Cr) was used as control treatment. For interaction with barley roots, surface sterile seeds were germinated on sterile filter paper placed on water agar (5 seeds per replicate). *C. rosea* conidia (1e + 07) were inoculated five days post germination and were allowed to interact for five days before harvesting of roots along with fungal mycelium. Harvested samples were immediately frozen in liquid nitrogen and stored at -80°C.

RNA extraction from all samples was done using the Qiagen RNeasy kit following the manufacturer’s protocol (Qiagen, Hilden, Germany). RNA was treated with RNase-free DNaseI (Fermentas, St. Leon-Rot, Germany) and concentrations were determined spectrophotometrically using NanoDrop (Thermo Scientific, Wilmington, DE). One or two microgram of total RNA was reverse transcribed in a total volume of 20 μl using the Maxima first stand cDNA synthesis kit (Fermentas, St. Leon-Rot, Germany). Transcript levels were quantified by qPCR using the SYBR Green PCR Master Mix (Fermentas, St. Leon-Rot, Germany) in an iQ5 qPCR System (Bio-Rad, Hercules, CA) as described previously [50]. Melt curve analysis was performed after the qPCR reactions, to confirm that the signal was the result from a single product amplification. Relative expression levels for target genes in relation to tubulin expression [51] were calculated from the Ct values and the primer amplification efficiencies by using the formula described by Pfaffl [52]. Gene expression analysis was carried out in 3 biological replicates, each based on 2 technical replicates. Primer sequences used for gene expression analysis are given in Additional file 1: Table S2.

### Construction of disruption and complementation vectors

Genomic DNA was isolated using a hexadecyltrimethylammonium bromide (CTAB)-based method [53]. Phusion DNA polymerase (Finnzymes, Vantaa, Finland) was used for PCR amplification of a 1 kb 5′-flank and 3′-flank region of the *Hyd1*, *Hyd2* and *Hyd3* genes from genomic DNA of *C. rosea* using primer pairs Hyd1 ko-1 F/1R and Hyd1 ko-2 F/2R; Hyd2 ko-1 F/1R and Hyd2 ko-2 F/2R; and Hyd3 ko-1 F/1R and Hyd3 ko-2 F/2R, respectively (Additional file 1: Table S2). The hygromycin resistance gene (*hygB*) cassette was amplified from the pCT74 vector [54] using the P3/P4 primer pair (Additional file 1: Table S2). The nourseothricin resistance gene (*nat1*) cassette was amplified from the pD-NAT1 vector [55] using the NatF/NatR primer pair (Additional file 1: Table S2). Gateway entry clones of the purified 5′-flank, 3′-flank, *hygB* and *nat* cassettes PCR fragments were generated as described by the manufacturer (Invitrogen, Carlsbad, CA). The gateway LR recombination reactions were performed using entry plasmid of respective fragments and destination vector pPm43GW [56] to generate the disruption vectors following the conditions described by the manufacturer (Invitrogen, Carlsbad, CA).

*Hyd1* and *Hyd3* complementation cassettes were constructed by PCR amplification of the full-length sequence of *Hyd1* and *Hyd3* including 1 kb upstream and downstream regions from genomic DNA of *C. rosea* WT using Hyd1 comp-F/R and Hyd3 comp-F/R primers, respectively (Additional file 1: Table S2). The amplified DNA fragments were purified and integrated into destination vector pPm43GW as described above using Gateway cloning technology to generate complementation vectors.

### *Agrobacterium tumefaciens* mediated transformation

The disruption and complementation vectors were transformed into *A. tumefaciens* strain AGL-1 as described before [31-33]. *A. tumefaciens* mediated transformation (ATMT) was performed based on a previous protocol [57]. Transformed strains were selected on plates containing hygromycin or nourseothricin or both in the case of double deletion and complementation experiment. Putative transformants were repeatedly sub-cultured on PDA plates without the selectable agent five times, followed by re-exposure to hygromycin or nourseothricin respectively, in order to test for mitotic stability. Mitotically stable colonies were purified by two rounds of single spore isolation.

### Validation of transformants

A PCR screening approach of putative transformants was performed to validate the homologous integration of the disruption cassette [31-33]. The primers used were specific to the *hygB* gene (P3/P4), sequences flanking the deletion construct (Hyd1-ups/ds for Δ*Hyd1*; and Hyd3-ups/ds for Δ*Hyd3*) and in combination (Hyd1-ups/HygR_qPCR, Hyd1-ds/HygF_qPCR for Δ*Hyd1;* and Hyd3-ups/HygR_qPCR, Hyd3-ds/HygF_qPCR for Δ*Hyd3*). Reverse transcriptase (RT-) PCR analysis was conducted on WT, deletion and complemented strains using RevertAid premium reverse transcriptase (Fermentas, St. Leon-Rot, Germany) and primer pairs specific for *hygB* (HygF_qPCR/HygR_qPCR), *nat1* (NatF_qPCR/NatR_qPCR), *Hyd1* (Hyd1-F/R) and *Hyd3* (Hyd3-F/R) (Additional file 1: Table S2).

### Phenotypic analysis

A 3 mm agar plug from the growing mycelial front was transferred to solid PDA, or PDA plates containing NaCl (0.5 M), sorbitol (1.5 M), SDS (0.05%) or caffeine (0.2%) in the case of abiotic stress analysis. Colony diameter was measured after 5 day of growth at 25°C. Conidiation rate was determined by harvesting spores from 10 day old PDA plate cultures using a Bright-Line haemocytometer (Sigma-Aldrich, St. Louis, MO) as per instruction. For conidial susceptibility assay, conidia were harvested in sterile water from the surface of two week old PDA plates, spread on PDA plates containing NaCl (0.5 M), sorbitol (1.5 M) or caffeine (0.2%). Conidia spread on only PDA plates served as control. For cold stress experiments, conidia at a concentration of 1e + 06 ml^-1^ in sterile water was incubated at 4°C for 3 days, 6 days or 9 days and then spread on PDA plates. Frequency of conidial germination was determined post 16 h of spreading by counting the number of germinating and non-germinating conidia using microscope. Two hundred to three hundred conidia were counted for each treatment. Each experiment had 3 biological replicates and was repeated 2 times.

Mycelial hydrophobicity of *C. rosea* strains were assayed on PDA plates post 3 days or 10 days of inoculation using water or SDS following the procedure described before [34]. The hydrophobicity of conidia was assayed using MATH [34], and hydrophobic index was calculated following the formula described before [10]. For extracellular protein concentration determination, fungal strains were grown for 10 days in liquid PDB medium at 25°C, mycelial debris were removed by filtering through four layers of Miracloth, followed by protein precipitation using an acetone precipitation protocol as described elsewhere. The protein pellets were dissolved in water and total extracellular protein concentration was determined using the quick start Bradford protein assay kit following the manufacturer’s instruction (Bio-Rad, Hercules, CA).

### Antagonism test

Antagonistic behaviour against phytopathogenic fungi *B. cinerea*, *F. graminearum* and *R. solani* was tested using an *in vitro* plate confrontation assay on PDA medium. An agar plug of *C. rosea* was inoculated 2 cm from the edge in a 9 cm PDA plate. After 7 days of incubation at 25°C, a plug of *B. cinerea*, *F. graminearum* or *R. solani* was placed at equal distance to the opposite edge of plate. To test the tolerance of *C. rosea* WT, deletion or complemented strains against secreted factors of *B. cinerea*, *F. graminearum* and *R. solani*, agar plugs of phytopathogenic fungi were inoculated on PDA plates covered with cellophane and incubated at 25°C in darkness. The plates covered with cellophane, without inoculation, were used as control. The cellophane was removed when fungal mycelia covered the plates, followed by inoculation with *C. rosea* WT, deletion or complementation strains. Linear growth was recorded daily in 3 replicates. For secretion assay, *C. rosea* strains were grown for 10 days in liquid PDB medium on rotary shaker at 25°C. Culture filtrate was collected after removing mycelia by filtering through four layers of Miracloth. The filtrate was further purified by passing through a 0.45 μM pore size nylon membrane. Agar plugs of *B. cinerea*, *F. graminearum* or *R. solani* was inoculated in conical flasks (50 ml) with 20 ml culture filtrate and incubated at 25°C under constant shaking condition (100 rpm). Biomass production in culture filtrates was analysed by determining mycelial dry weight post 3 days of inoculation.

### Detached leaf bioassay

*B. cinerea* conidia were collected from 15 days old PDA plates with distilled water and filtered to remove the mycelial debris. Four leaves of 3-week-old *A. thaliana* ecotype Colombia-0 (Col-0) plants, grown in a Percival growth chamber (CLF plant climates, GmbH, Germany) with growth conditions described before [32,33], were detached from each plant and placed on water agar plate with petiole inserted in agar. A 5 μl droplet of conidial suspension (1e + 06 conidia ml^−1^) of *C. rosea* WT, deletion or complemented strains were inoculated on the adaxial surface of the leaf, dried for 30 min and re-inoculated with equal conidial concentration of *B. cinerea* at the same place. Plants were kept in Percival growth chambers and high humidity was maintained by sealing the plates with parafilm. The diameter of necrotic lesions was measured post 56 h of inoculation under the microscope using a DeltaPix camera and software (DeltaPix, Denmark). Bioassay experiments were performed in 3 biological replicates and each replicate consisted of 16 leaves from 4 plants for each treatment. The experiment was repeated 2 times.

### *Arabidopsis thaliana* root colonization assay

Surface sterile seeds of *A. thaliana* ecotype Col-0 were grown on 0.2X MS agar plates. Plates were settled vertically, to avoid burial of roots in medium, in a Percival growth chamber (CLF plant climates, GmbH, Germany) with a growth conditions described before [32,33]. *C. rosea* conidia (5e + 04) were inoculated under sterile conditions to the middle of 10 days old seedling roots and were co-cultivated for 5 days. Water inoculated roots were treated as control. For each set of experiments 5 biological replicates with 10 seedlings per replicate were used. To quantify the root colonization, detached roots were washed carefully with water, surface sterilized with 2% NaOCl for 1 min, weighed, and homogenised in 2 ml sterile water. Serial dilutions were plated on PDA plates to count colony forming units. The complementation strains Δ*Hyd1+* and Δ*Hyd3+* and four independent *Hyd1Hyd3* mutant strains were included in all phenotype analyses to exclude the possibility that phenotypes derive from ectopic insertions. No significant difference in data of analysed phenotypes were found between four independent *Hyd1Hyd3* mutant strains, therefore data from one representative deletion strain are presented in the figures.

### Statistical analysis

Analysis of variance (ANOVA) was performed on gene expression and phenotype data using a General Linear Model approach implemented in Statistica version 10 (StatSoft, Tulsa, OK). Pairwise comparisons were made using the Tukey-Kramer method at the 95% significance level.

## Competing interests

The authors declare that they have no competing interests.

## Authors’ contributions

Conceived and designed the experiments: MD DFJ MK. Performed the experiments: MD. Analyzed the data: MD MK. Contributed reagents/materials/analysis tools: DFJ MK. Wrote the paper: MD MK. All authors read and approved the manuscript.

## Supplementary Material

Additional file 1: Table S1 Protein Genbank accession number for class II hydrophobin sequences used for phylogenetic tree construction and alignment. **Table S2.** List of primers used in this study. **Figure S1.** Gene expression analysis during different stages of interaction with B. cinerea (Cr-Bc) or F. graminearum (Cr-Fg). **Figure S2.** Schematic representation of deletion cassettes and characterization of mutant strains using PCR and RT-PCR. **Figure S3.** The Δ*Hyd1*Δ*Hyd3* mutant showed reduced conidial surface hydrophobicity. **Figure S4.** Tolerance of C. rosea strains mycelia to abiotic stress. **Figure S5.** Expression analysis of Hyd2 in C. rosea WT, Δ*Hyd1*, Δ*Hyd3* and Δ*Hyd1*Δ*Hyd3* mutant strains. Figure legends to additional figures are described in detail in introduction section of additional file. Click here for file
