# Supplementary material for: Hydrophobins are required for conidial hydrophobicity and plant root colonization in the fungal biocontrol agent Clonostachys rosea
Source: BMC Microbiol. 2014 Jan 31;14:18. doi: 10.1186/1471-2180-14-18 (PMC3922079; doi:10.1186/1471-2180-14-18)
Supplement: Additional file 1: Table S1 — Protein Genbank accession number for class II hydrophobin sequences used for phylogenetic tree construction and alignment. Table S2. List of primers used in this study. Figure S1. Gene expression analysis during different stages of interaction with B. cinerea (Cr-Bc) or F. graminearum (Cr-Fg). Figure S2. Schematic representation of deletion cassettes and characterization of mutant strains using PCR and RT-PCR. Figure S3. The ΔHyd1ΔHyd3 mutant showed reduced conidial surface hydrophobicity. Figure S4. Tolerance of C. rosea strains mycelia to abiotic stress. Figure S5. Expression analysis of Hyd2 in C. rosea WT, ΔHyd1, ΔHyd3 and ΔHyd1ΔHyd3 mutant strains. Figure legends to additional figures are described in detail in introduction section of additional file. [file 1471-2180-14-18-S1.pdf]

Additional File; Table S1

Table S1: Protein GenBank accession numbers for class II hydrophobin sequences used for phylogenetic tree construction and alignment.

**Organisms name**	**Abbreviation**	**GenBank accession number**
Aspergillus terreus	A_TER	XP_001213908.1
Claviceps purpurea	CLF	Q9UVI4.1
C. purpurea	CLP	CAD10781.1
Cryphonectria parasitica	CRP	P52753.1
Fusarium graminearum	GIZ	XP_382007.1
F. moniliforme	GIM	AAO16870.1
Magnaporthe oryzae	MGG1	XP_363247.1
M. oryzae	MGG2	XP_364289.1
Neurospora crassa	NEC	XP_959282.2
Passalora fulva	PF1	CAC27407.1
P. fulva	PF2	CAB39312.1
Trichoderma atroviride	HA_1b	ABS59362.1
T. atroviride	HA_1c	ABS59363.1
T. atroviride	HA_2a	CAA72539.1
T. atroviride	HA_2b	ABS59365.1
T. atroviride	HA_2c	ABS59371.1
T. atroviride	HA_5a	ABS59366.1
T. atroviride	HA_6a	ABS59367.1
T. atroviride	HA_6b	ABS59368.1

T. atroviride	HA_6c	ABS59369.1
T. atroviride	HA_22a	ABS59370.1
T.virens	HV_1a	ABS59372.1
T.virens	HV_1b	ABS59373.1
T.virens	HV_1c	ABS59374.1
T.virens	HV_1d	ABS59375.1
T.virens	HV_2a	ABS59376.1
T.virens	HV_13a	ABS59377.1
T.virens	HV_18a	ABS59378.1
T.virens	HV_21a	ABS59380.1
T.virens	HV_22a	ABS59379.1
T. aggresivum var. *europeae*	TAE_1	ES768856)
T. aggresivum var. *europeae*	TAE_2	ES768855)
T. aggresivum var. *europeae*	TA_1	AJ903054)
T. asperellum	TA_3	AJ902899)
T. asperellum	TA_4	AJ903666
T. viride	TCK_1	AJ909436
T. viride	TCK_2	EV554903
T. longibrachiatum	TL_1	AJ905782
T. reesei	HFB1	P52754.1
T. reesei	HFB2	P79073.1
T. harzianum	HL_1	AJ896766
T. harzianum	HL_2	AJ896364
T. harzianum	HL_3	AJ897108

T. harzianum	HL_4	ABN64104
T. harzianum	QID3	X71913.1)
Verticillium dahliae	VED	AAY89101.1
Pleurotus ostreatus	POH2	CAA74987.1
P. ostreatus	POH3	CAA76494.1

Additional File; Table S2

Table S2: List of primers used in this study

Primer name	Target gene	Sequences (5´ → 3`)
Cr-1F	*Hyd1*	ctc tcc acc cat tct acg gtt tc
Cr-1R		aaa tat cg gaga agt aga gaa cgc
Cr-2F	*Hyd2*	act cca ctg cat ttc aca tca ctc
Cr-2R		gag ttc tca agc ggc atc aaa g
Cr-3F	*Hyd3*	ctt tcg ctt cat ctc atc tca gtt
Cr-3R		aag ggg cga agg caa gac a
Hyd1-cds-F	*Hyd1*	tga agt tta tcg cta cca cca t
Hyd1-cds-R		tca ccg gcc tac gag aat a
Hyd2-cds-F	*Hyd2*	atg aga tct ttc ctt gtt att gcg
Hyd2-cds-R		ggt tca gga cct cgt tga gg
Hyd3-cds-F	*Hyd3*	ttc tag cta ccg ctg ctc tcc t
Hyd3-cds-R		aag cac ata gat cga gcc caa
Hyd1-F	*Hyd1*	ctt gcc gct cgc ctc ttt tc
Hyd1-R		agg ggg atg gat caa caa ctc a
Hyd2-F	*Hyd2*	aac cgg atc gta ccc caa gt
Hyd2-R		at cgc cct cag ggt taa atg tat
Hyd3-F	*Hyd3*	cgg caa agc gtc aga cat aca t
Hyd3-R		caa gtt tac gag gcc caa gac g
Cr-tub F		ttc aga ccg gtc agt gcg ta

Cr-tub R	*tubulin*	gcc aga aag cag ca caa t
Hyd1 ko-1F	*Hyd1*	[1]gggg aca act ttg tat aga aaa gtt g: ata gcg tgc act tgc ctt ctc a
Hyd1 ko-1R		[1]gggg ac tgc ttt ttt gta caa act tg: gac gag aat ggt ggt agc gat aaa
Hyd1 ko-2F		[1]gggg aca gct ttc ttg tac aaa gtg g: gcc ggt gat agt ggc ttt gaa
Hyd1 ko-2R		[1]gggg ac aac ttt gta taa taa agt tg: ggc cgt cct ttg ctg cta at
Hyd2 ko-1F	*Hyd2*	[1]gggg aca act ttg tat aga aaa gtt g: aaa taa tgt caa ggg cgg gaa t
Hyd2 ko-1R		[1]gggg ac tgc ttt ttt gta caa act tg: tgc tta aag agt gat gtg aaa tgc
Hyd2 ko-2F		[1]gggg aca gct ttc ttg tac aaa gtg g: gtt gct ttg atg ccg ctt ga
Hyd2 ko-2R		[1]gggg aca gct ttc ttg tac aaa gtg g: gaa att cgg tcc aag cac tga g
Hyd3 ko-1F	*Hyd3*	[1]gggg aca act ttg tat aga aaa gtt g:agg ggg ctt gtt cct ctc ca
Hyd3 ko-1R		[1]gggg ac tgc ttt ttt gta caa act tg:tcg ggc gag gtt gat ttt tat t
Hyd3 ko-2F		[1]gggg aca gct ttc ttg tac aaa gtg g:ttg cct tcg ccc ctt gag at
Hyd3 ko-2R		[1]gggg ac aac ttt gta taa taa agt tg:acc tgc cgg ctg cta tca act
P3	*hph*	[1]gggg aca agt ttg tac aaa aaa gca ggc t:gca ccc cgc tat aac tga cg
P4		[1]gggg ac cac ttt gta caa gaa agc tgg gt:gcg cgc aat taa ccc tca c
Nat F	*nat1*	[1]gggg aca agt ttg tac aaa aaa gca ggc tta:cag aat tcg tga tga att
Nat R		[1]gggg ac cac ttt gta caa gaa agc tgg gta:gac gaa ttc aga tgg gcc
Nat1F	*nat1*	ggc tgg agc tag tgg agg tca aca
Nat1R		gtg ctc cgg ggc gac ctc
NatF_qPCR	*nat1*	gga ccc gcc cct gac caa
NatR_qPCR		gtg ctc cgg ggc gac ctc
Hyg F	*hph*	gcg cgc aat taa ccc tca c
Hyg R		gaa ttg cgc gta cag aac tcc
HygF_qPCR	*hph*	acg gcg gga gat gca ata ggt

HygR_qPCR		gct tcg atg tag gag ggc gtg g
Hyd1-ups	*Hyd1*	aac gtg cat gtc tgt att tgt gag
Hyd1-ds		gct tcg gag gca cat tta cac
Hyd3-ups	*Hyd3*	ttc tgg gac agg ttc ttg ctc t
Hyd3-ds		aac atg gac aat ctg cgt gaa at
Hyd1 comp-F	*Hyd1*	[1]gggg aca act ttg tat aga aaa gtt g:ata gcg tgc act tgc ctt ctc a
Hyd1 comp-R		[1]gggg ac tgc ttt ttt gta caa act tg:ggc cgt cct ttg ctg cta at
Hyd3 comp-F	*Hyd3*	[1]gggg aca act ttg tat aga aaa gtt g:agg ggg ctt gtt cct ctc ca
Hyd3 comp-R		[1]gggg ac tgc ttt ttt gta caa act tg:acc tgc cgg ctg cta tca act

[1]attB and attBr sequences are underlined;

Abbreviation used for enzyme encoding genes:

hph=hygromycin B phosphotransferase; *nat1*=Nouresothricin N-acetyltransferase;

Hyd=Hydrophobin

Figure S1

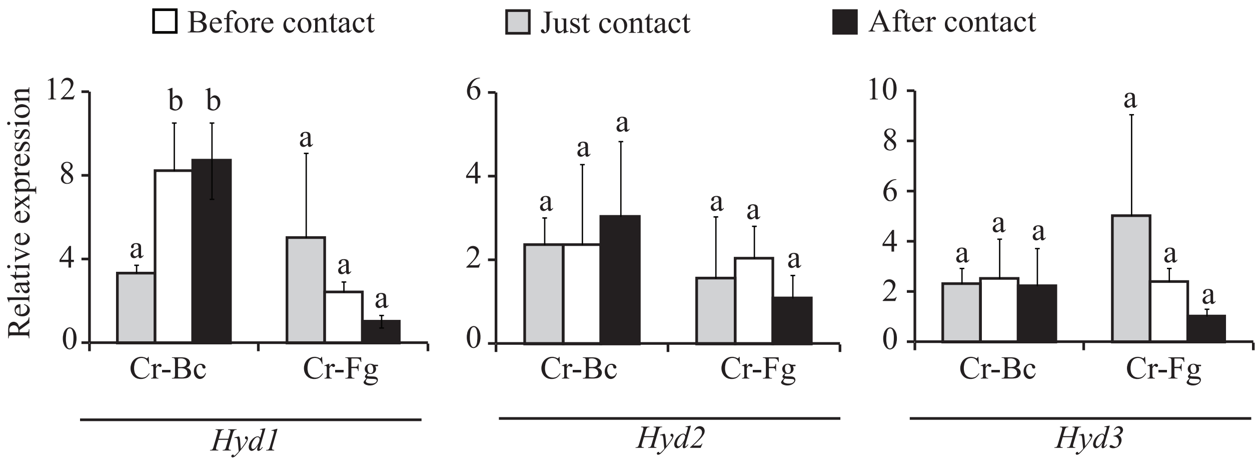

Figure S1. Gene expression analysis during different stages of interaction with *B. cinerea* (Cr-Bc) or *F. graminearum* (Cr-Fg). Expression levels for *Hyd1*, *Hyd2* and *Hyd3* was normalized by tubulin expression, using the formula described by Pfaffl [51]. Error bars represent standard deviation based on 3 biological replicates. Different letters indicate statistically significant differences ($P \leq 0.05$) within experiments based on the Tukey-Kramer test.

Additional File; Figure S2

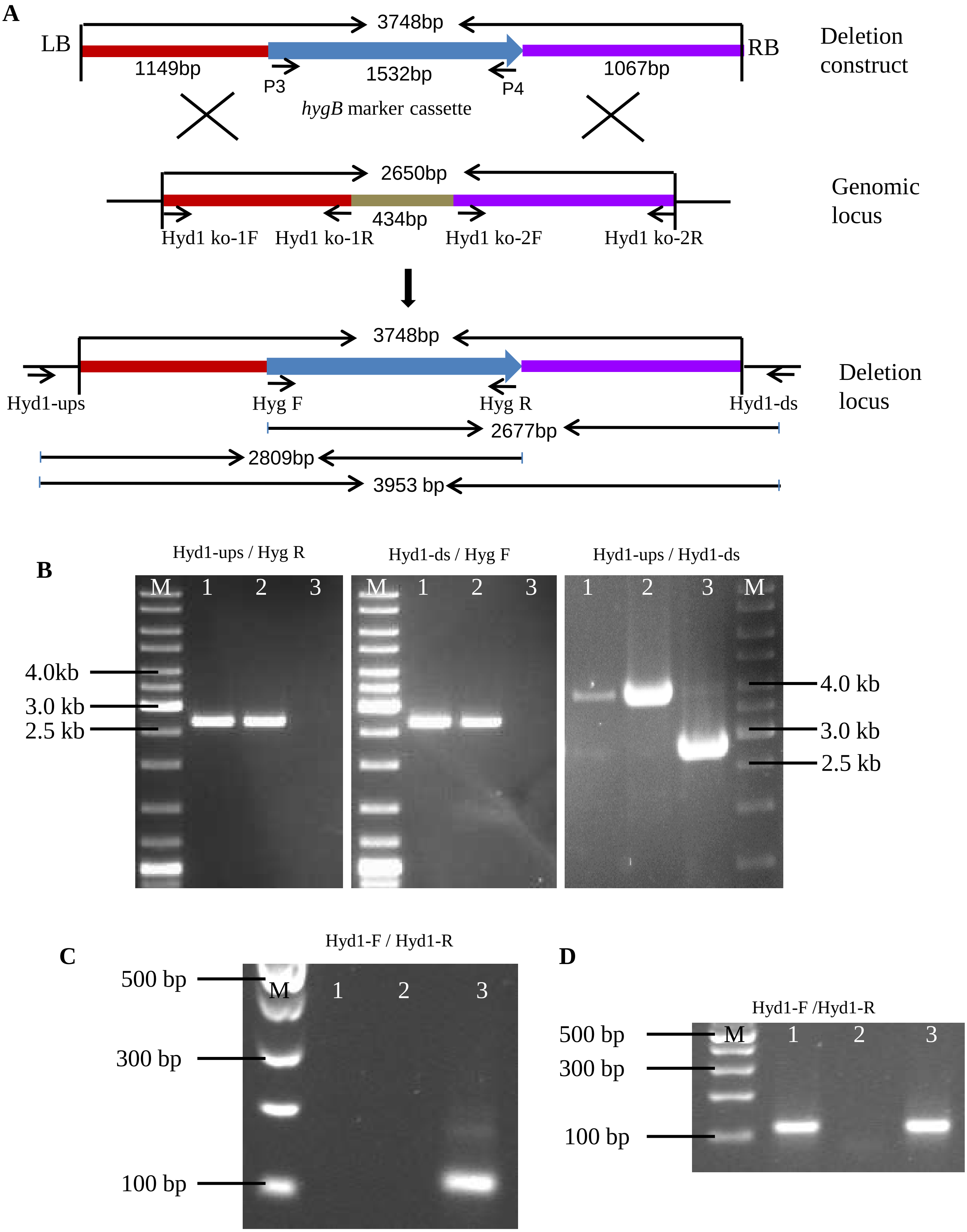

E

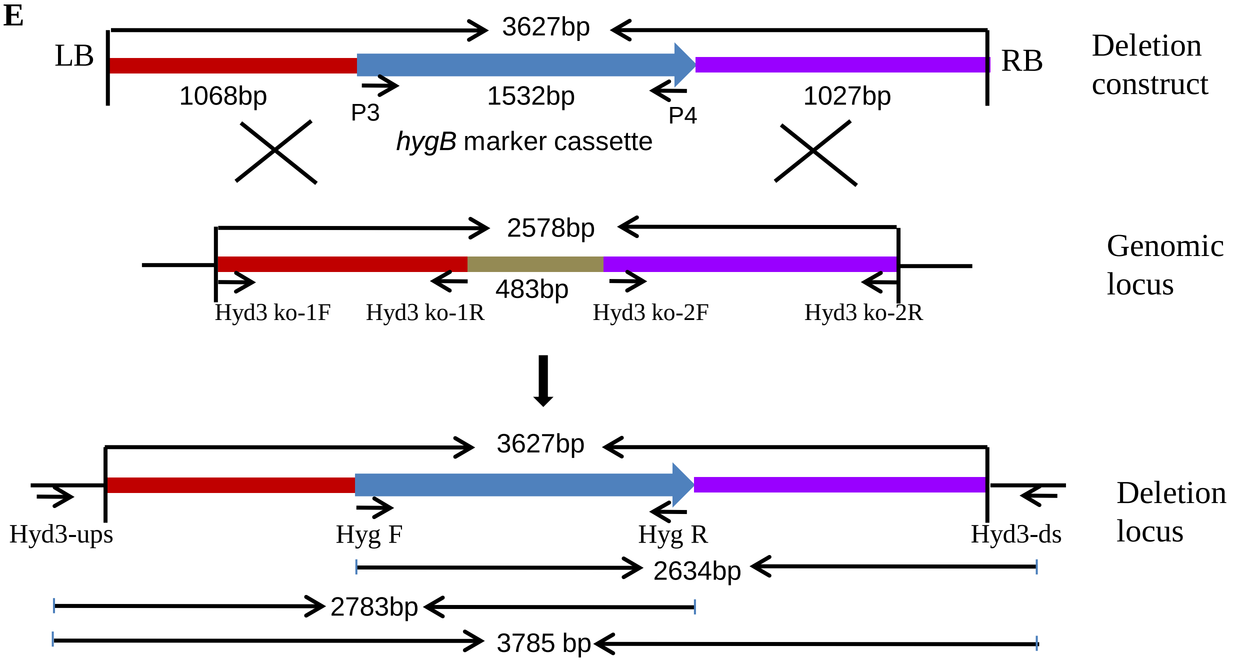
3627bp
LB
RB
1068bp
1532bp
1027bp
P3
P4
hygB marker cassette
Deletion construct
2578bp
483bp
Hyd3 ko-1F
Hyd3 ko-1R
Hyd3 ko-2F
Hyd3 ko-2R
Genomic locus
3627bp
Hyd3-ups
Hyg F
Hyg R
Hyd3-ds
Deletion locus
2634bp
2783bp
3785 bp

F

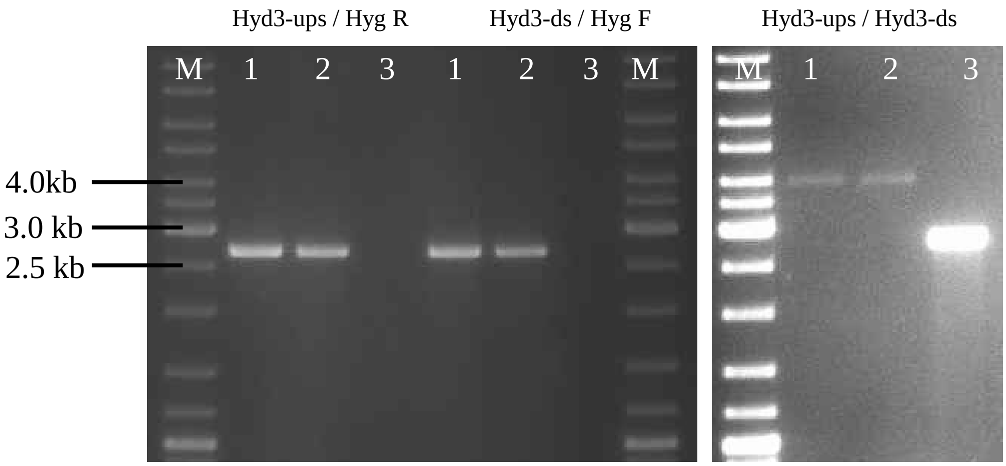
Hyd3-ups / Hyg R
Hyd3-ds / Hyg F
Hyd3-ups / Hyd3-ds
M 1 2 3 1 2 3 M
M 1 2 3
4.0kb
3.0 kb
2.5 kb

G

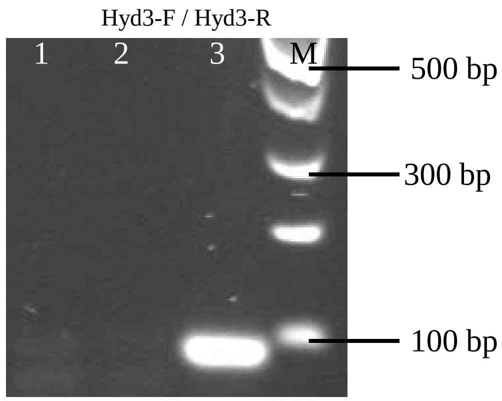
Hyd3-F / Hyd3-R
1 2 3 M
500 bp
300 bp
100 bp

H

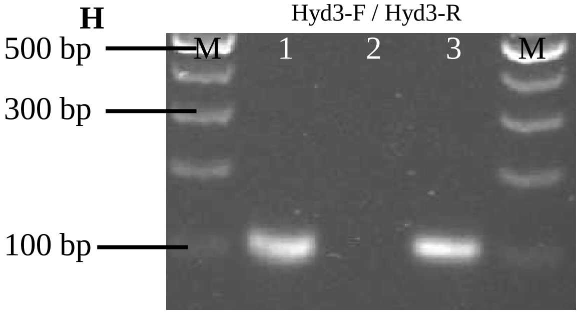
Hyd3-F / Hyd3-R
M 1 2 3 M
500 bp
300 bp
100 bp

I

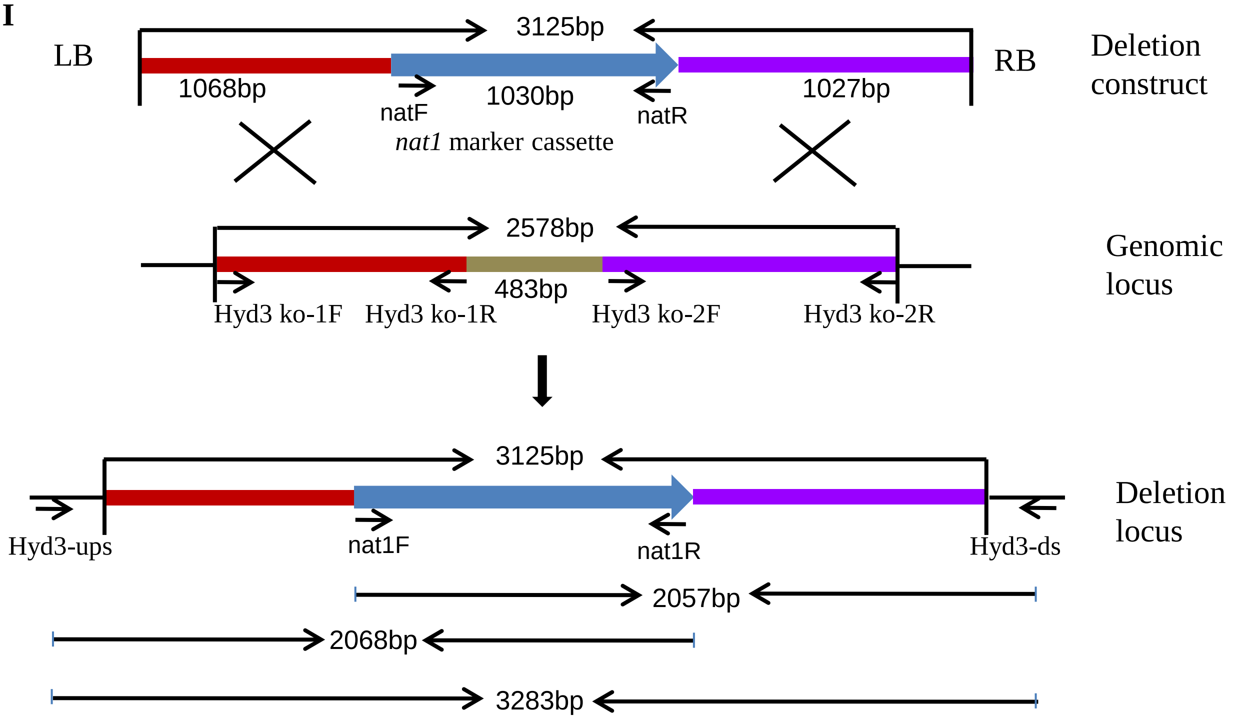

LB
3125bp
RB
Deletion construct
1068bp
natF
1030bp
natR
1027bp
nat1 marker cassette
2578bp
Genomic locus
483bp
Hyd3 ko-1F
Hyd3 ko-1R
Hyd3 ko-2F
Hyd3 ko-2R
3125bp
Deletion locus
Hyd3-ups
nat1F
nat1R
Hyd3-ds
2057bp
2068bp
3283bp

J

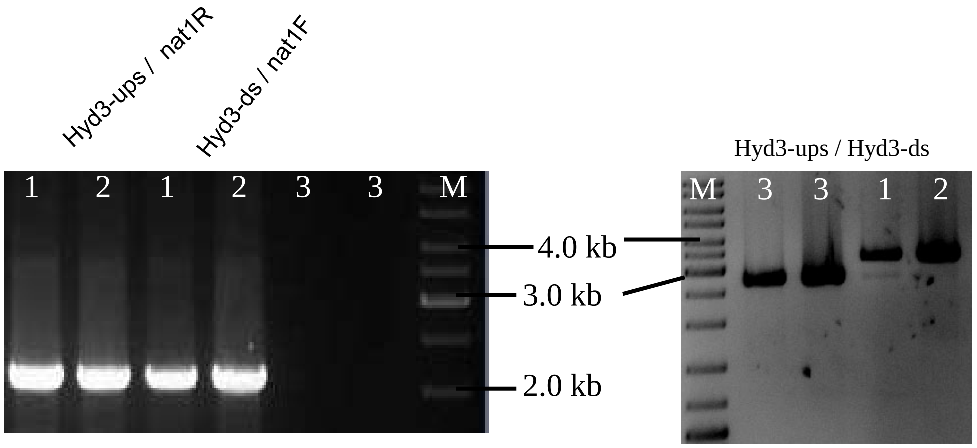

Hyd3-ups / nat1R
Hyd3-ds / nat1F
1 2 1 2 3 3 M
4.0 kb
3.0 kb
2.0 kb
Hyd3-ups / Hyd3-ds
M 3 3 1 2

K

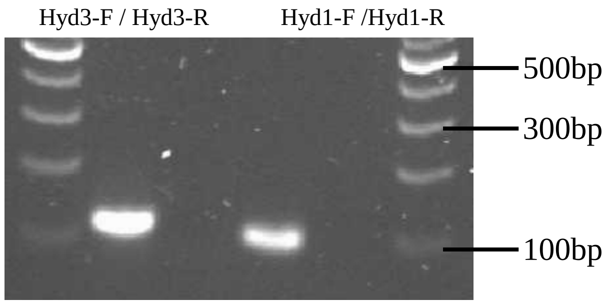

Hyd3-F / Hyd3-R
Hyd1-F /Hyd1-R
500bp
300bp
100bp

Figure S2. Schematic representation of deletion cassettes and characterization of mutant strains using PCR and RT-PCR.

A: Organisation of *Hyd1* locus in WT and mutant strain of *C. rosea* strain. The *Hyd1* coding region was replaced by *hygB* cassette by homologous recombination resulting in generation of Δ*Hyd1* strains. The small arrow heads indicate the location of primers used to construct the deletion cassette and analysis of mutants using PCR. The large arrow heads indicate the size of amplified PCR products. Abbreviations: LB, left border; RB, right border. **B:** PCR verification of Δ*Hyd1* strain using primers located in the *hygB* gene (Hyg F/Hyg R) and upstream or/and downstream from the deletion cassette (Hyd1-ups/Hyd1-ds). PCR products of ~2.8 kb and ~2.7 kb using primers Hyd1-ups/ Hyg R and Hyd1-ds/ Hyg F, respectively were expected from a correct gene replacement in Δ*Hyd1* strains. PCR product of ~4.0 kb and ~2.8 kb using primers Hyd1-ups/ Hyd1-ds were expected from the Δ*Hyd1* and WT strains, respectively.

C: RT-PCR analysis of gene expression in Δ*Hyd1* and WT strains, using *Hyd1* specific Hyd1-F/Hyd1-R primers.

M, gene ruler DNA ladder mix; 1, 2, independent Δ*Hyd1*mutant; 3, WT strains

D: Validation of complementation strains. RT-PCR analysis of *Hyd1* expression in WT, Δ*Hyd1*, and Δ*Hyd1*+ complemented strains, using *Hyd1* specific Hyd1-F/Hyd1-R primers. M, gene ruler DNA ladder mix; 1, WT; 2, Δ*Hyd1*; 3, Δ*Hyd1*+ strains

Primer combinations used for PCR and RT-PCR are given above the images.

E: Organisation of *Hyd3* locus in WT and mutant strain of *C. rosea* strain. The *Hyd3* coding region was replaced by *hygB* cassette by homologous recombination resulting in generation of Δ*Hyd3* strains. The small arrow heads indicate the location of primers used to construct the deletion cassette and analysis of mutants using PCR. The large arrow heads indicate the size of amplified PCR products. Abbreviations: LB, left border; RB, right border.

F: PCR verification of Δ*Hyd3*strain using primers located in the *hygB* gene (Hyg F/Hyg R) and upstream or/and downstream from the deletion cassette (Hyd3-ups/Hyd3-ds). PCR products of ~2.8 kb and ~2.7 kb using primers Hyd3-ups/ Hyg R and Hyd3-ds/ Hyg F, respectively were expected from a correct gene replacement in Δ*Hyd3* strains. PCR product of ~3.8 kb and ~2.8 kb using primers Hyd3-ups/ Hyd3-ds were expected from the Δ*Hyd3* and WT strains, respectively.

G: RT-PCR analysis of gene expression in Δ*Hyd3* and WT strains, using *Hyd3* specific Hyd3-F/Hyd3-R primers.

M, gene ruler DNA ladder mix; 1, 2, independent Δ*Hyd3* mutant; 3, WT strains

H: Validation of complementation strains. RT-PCR analysis of *Hyd3* expression in WT, Δ*Hyd3*, and Δ*Hyd3*+ complemented strains, using *Hyd3* specific Hyd3-F/Hyd3-R primers.

M, gene ruler DNA ladder mix; 1, WT; 2, Δ*Hyd3*; 3, Δ*Hyd3*+ strains

Primer combinations used for PCR and RT-PCR are given above the images.

I: Construction of deletion cassette using *nat1* selection marker for double deletion mutants. The *Hyd3* coding region was replaced by *nat1* cassette by homologous recombination in Δ*Hyd1* resulting in generation of Δ*Hyd1*Δ*Hyd3* deletion mutant. The small arrow heads indicate the location of primers used to construct the deletion cassette and analysis of mutants using PCR. The large arrow heads indicate the size of amplified PCR products. Abbreviations: LB, left boarder; RB, right boarder.

J: PCR verification of Δ*Hyd1*Δ*Hyd3* using primers located in the *nat1* gene (nat 1F/nat 1R) and upstream or/and downstream from the deletion cassette (Hyd3-ups/Hyd3-ds). PCR products of ~2.1 kb and ~2.1 kb using primers Hyd3-ups/ nat 1R and Hyd3-ds/ nat 1F, respectively were expected from a correct gene replacement in Δ*Hyd1*Δ*Hyd3* strains. PCR product of ~3.3 kb and ~2.7 kb using primers Hyd3-ups/ Hyd3-ds were expected from the Δ*Hyd1*Δ*Hyd3* and WT strains, respectively. M, gene ruler DNA ladder mix; 1, 2, independent Δ*Hyd1*Δ*Hyd3*; 3 WT strains.

K: RT-PCR analysis of *Hyd1* and *Hyd3* expression in Δ*Hyd1*Δ*Hyd3* and WT strains, using *Hyd1* and *Hyd3* specific primers Hyd1-F/Hyd1-R and Hyd3-F/Hyd3-R, respectively.

M, gene ruler DNA ladder mix; 1, WT strains; 2, independent Δ*Hyd1*Δ*Hyd3* mutant.

Additional File; Figure S3

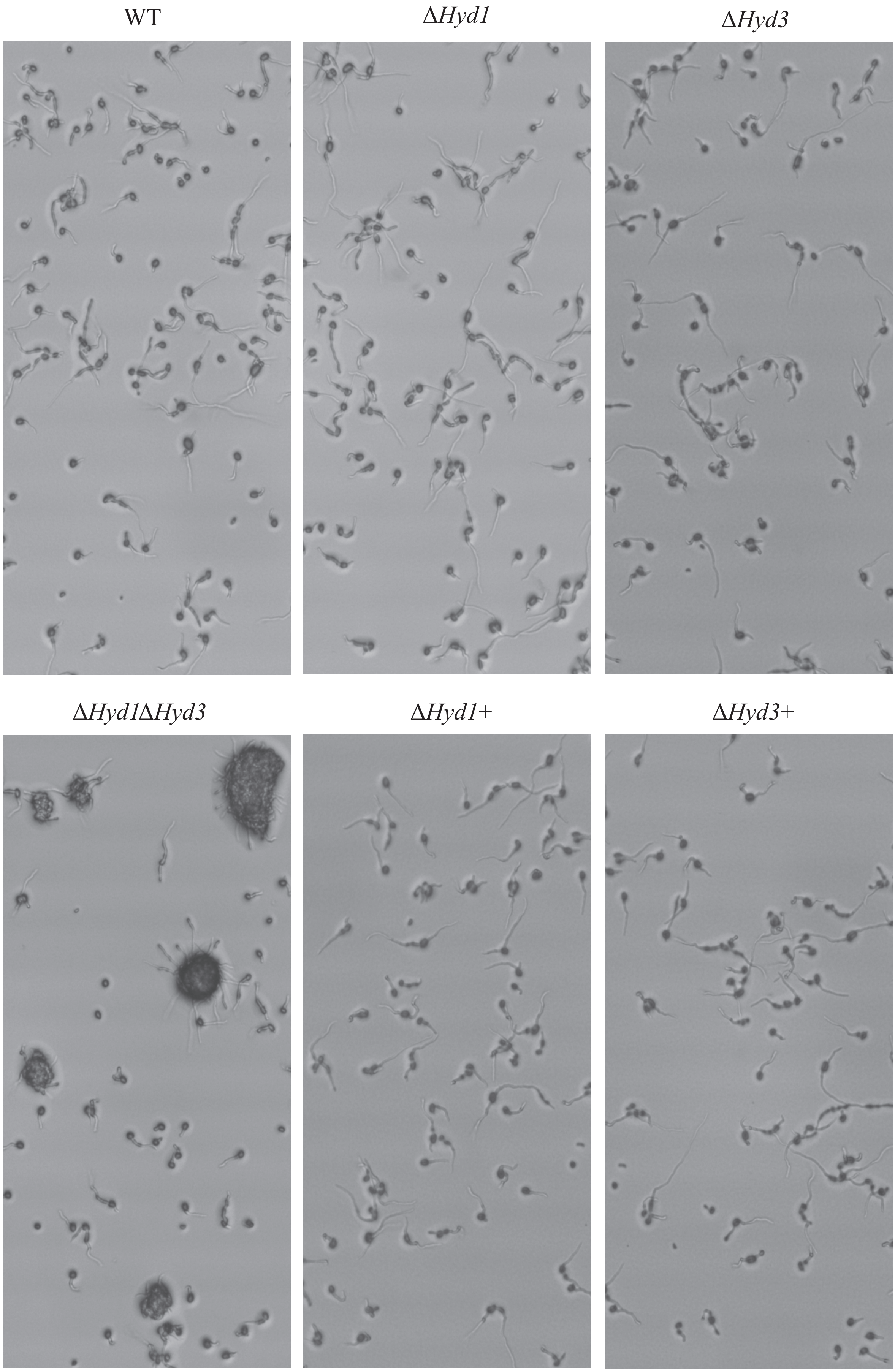

Figure S3: The *ΔHyd1ΔHyd3* mutant showed reduced conidial surface hydrophobicity. Images of **c**onidial suspension of *C. rosea* strains harvested in water from 10 days old cultures grown on solid agar.

Additional File; Figure S4

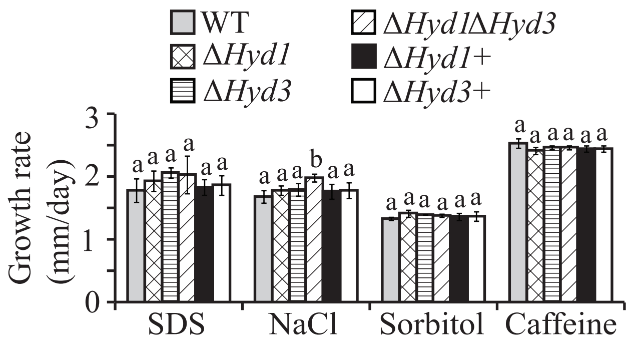

Figure S4: **Tolerance of *C. rosea* strains mycelia to abiotic stress.** Strains were inoculated on solid PDA plates supplemented with NaCl (0.5M), sorbitol (1.5M), SDS (0.05%) or caffeine (0.2%) and incubated at 25°C in darkness. Growth rate was calculated from data recorded 5 days post inoculation. Error bars represent standard deviation based on three biological replicates. Different letters indicate statistical significance ($P \leq 0.05$) for strain differences within a single medium.

Additional File; Figure S5

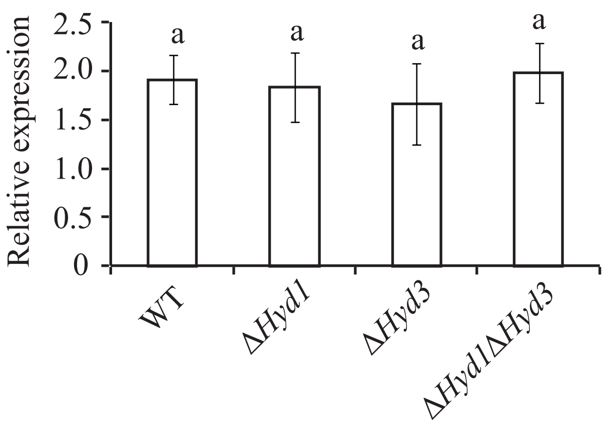

Figure S5: Expression analysis of *Hyd2* in *C. rosea* WT, Δ*Hyd1*, Δ*Hyd3* and Δ*Hyd1*Δ*Hyd3* mutant strains. Total RNA extracted from the conidiated mycelium (10 days post inoculation on PDA) of WT, Δ*Hyd1*, Δ*Hyd3* and Δ*Hyd1*Δ*Hyd3* mutant strains was used as template for cDNA synthesis. Expression levels were normalized by tubulin expression, using the formula described by Pfaffl [51]. Error bars represent standard deviation based on 4 biological replicates. Same letters indicate no statistically significant differences ($P \leq 0.05$) based on the Tukey-Kramer test.
